# Healing the Sole: Combining Natural Plant Extracts and Conventional Drugs in Wound Delivery Systems for Diabetic Foot Ulcer Treatment

**DOI:** 10.3390/ijms27156997

**Published:** 2026-08-04

**Authors:** Raneille Quirsten Baligod, Izliah Grace Nicole Fabian, Kristine Margreth Repol, Heidi Lindt Sy, Jubert Marquez, Joseph Rey Sta. Agueda

**Affiliations:** 1 Department of Biomedical, Manufacturing, and Robotics Engineering, De La Salle University, 2401 Taft Avenue, Malate, Manila 1004, Philippines; 2 Department of Biology, De La Salle University, 2401 Taft Avenue, Malate, Manila 1004, Philippines; 3 Basic Research Laboratory, Department of Physiology, College of Medicine, Smart Marine Therapeutic Center, Cardiovascular and Metabolic Disease Core Research Center, Inje University, Busan 47392, Republic of Korea; 4 Center for Engineering and Sustainable Development Research, De La Salle University, 2401 Taft Avenue, Malate, Manila 1004, Philippines

**Keywords:** chronic wounds, bioactive dressings, wound healing, phytochemicals, drug delivery, biomaterials

## Abstract

Diabetic foot ulcers (DFUs) affect 19–34% of diabetic patients with a five-year mortality rate of 50–70%. It is characterized by a high infection risk, chronic inflammation, oxidative stress, and poor angiogenesis, rendering existing therapies inadequate, which causes antimicrobial resistance and amputations. Bioactive compounds, such as curcumin, flavonoids, tannins, polyphenols, and proteins, found in natural product extracts (NPEs) have antibacterial, anti-inflammatory, antioxidant, and regenerative properties but are prone to variability and instability. Conventional drugs can be applied for targeted infection management and metabolic modulation, but lack regenerative capacity. The combined and synergistic potential of these two agents has not received much attention, especially when integrated into advanced drug delivery systems (DDSs), such as microneedles, hydrogels, nanofibers, and smart bandages. This review discusses NPEs, conventional drugs, combinations, and integration into DDSs, based on wound characteristics. These include depth, infection status, exudate level, and perfusion, as recommended by the 2023 IWGDF guidelines. Three combinations have shown promise in preclinical studies: propolis-charcoal bandages, propolis-oregano microneedles, and Centella-ZnO nanocomposites. However, none have yet been validated in clinical settings, and barriers to clinical adoption persist—standardization, stability, and regulatory issues. This work aims to pave the way for more accessible, sustainable, and efficacious DFU therapeutics by bridging natural and conventional approaches.

## 1. Introduction

Diabetic Foot Ulcer (DFU) is an increasing global health concern, affecting 19–34% of patients with Diabetes Mellitus (DM). The five-year mortality rate after DFU diagnosis is 50–70%, which is worse than breast and prostate cancer [1,2]. Among hospitalized DFU patients, 56% require lower extremity amputations [2]. The normal wound healing follows a sequence of stages, including hemostasis, inflammation, proliferation, and remodeling [3]. With DM, patients experience chronic hyperglycemia, which reduces growth factors such as VEGF and TGF-β [2,4], while fibroblast dysfunction, excessive production of reactive oxygen species (ROS), and impaired angiogenesis further delay tissue repair and increase risk of infection [5,6,7]. Bacterial biofilms may form from infections, shielding pathogens from antibiotics, and contributing to low-grade infections that can progress to osteomyelitis [8,9]. Beyond physical burden, DFUs also impose severe negative psychosocial effects on patients, such as malodor, exudates, disfigurement, and chronic pain, leading to patient discomfort, social stigma, isolation, and depression [10,11,12,13].

Current DFU treatment involves a combination of wound cleaning and debridement, antibiotics, dressings, and glycemic control [14]. However, the standard of DFU care faces three fundamental challenges. First, it addresses individual pathology rather than multiple processes simultaneously. For instance, antibiotics do not promote angiogenesis, and dressings manage exudates but do not address oxidative stress [15,16]. Second, antimicrobial resistance (AMR) is compounded by the overuse of antibiotics, with *Methicillin-resistant Staphylococcus aureus* (MRSA), vancomycin-resistant enterococci, and extended-spectrum beta-lactamase-producing Gram-negatives now routine in DFU cultures [8,9,17]. Lastly, the wound is ischemic, hypoxic, and has an impaired healing response since debridement and existing dressings are unable to restore perfusion or stimulate angiogenesis [7,18]. These limitations demand new approaches.

Alternative solutions such as natural products, specialized antimicrobial dressings, or a combination of natural products and wound delivery systems may combat drug-resistant infections, reduce ROS levels, and aid neovascularization [17]. Natural product extracts (NPEs) are one of such alternatives, derived from plants, animals, and microorganisms [19]. Bioactive compounds such as flavonoids, polyphenols, terpenes, and glycosides found in NPEs, such as curcumin [20,21], propolis [22,23], and *Centella asiatica* [24,25], demonstrate anti-inflammatory, antimicrobial, antioxidant, and pro-healing properties, which improve DFU healing. However, variability in extract composition, lack of standardization, and short shelf life contribute to difficulties in clinical translation [10,26,27,28]. Thus, conventional antibacterials, antidiabetics, and wound-healing medications, whether topical or systemic, are also crucial for treating DFU [29]. Medications such as mupirocin are pure, potent, and predictable, which offer local infection control, metabolic regulation, and cell proliferation [30]. However, they are constrained by high costs, systemic toxicity, AMR, and even insufficient capacity for tissue regeneration [31,32].

Although both NPEs and conventional drugs are effective when applied individually, their combined effects have not been fully investigated. Hence, this review critically assesses the use of combining specific NPEs with conventional drugs in advanced wound DDSs and examines their biological activities, medicinal properties, and interactions with commonly available medications. The 2023 International Working Group on the Diabetic Foot (IWGDF) recommended that individual wound characteristics, such as depth, infection, ischemia, and area, be used to describe wounds rather than relying on total scores [33,34]. Following this principle, we propose a framework that matches combination therapies to these wound characteristics. The framework is provisional and hypothesis-generating, intended to inform future research design rather than direct clinical practice.

### Literature Search Strategy

A thorough literature search was conducted across four main databases: Scopus, PubMed, ScienceDirect, and Google Scholar. Combinations of keywords related to DFUs (“diabetic foot ulcer”, “DFU”, “chronic wounds”), NPEs (“natural product extracts”, “natural product”, “plant extracts”, “phytochemicals”), conventional drugs (“topical antibiotics”, “diabetic drugs”, “DFU antibiotics”), DDS (“drug delivery systems”, “bandages”, “wound dressings”), wound healing properties (“antioxidant”, “anti-inflammatory”, “antibiotic”, “angiogenic”, “cell proliferation”), and combination therapies (“combination”, “synergy”, “antagonism”, “interactions”). Boolean operators AND and OR were used to narrow down results. Only English-language, open-access, peer-reviewed articles from the last 20 years that included both preclinical (in vitro and in vivo) and clinical research were included in the search. Studies that examined the use of NPEs and conventional drugs in wound healing, either individually or combined, evaluated their antibacterial, antioxidative, and wound-healing properties, or evaluated drug delivery systems intended for wound treatment, especially DFU, were deemed qualified. Only pertinent, high-quality sources were included.

## 2. Standard of Care

Standard care for DFUs addresses both the wound and its underlying condition to reduce amputation rates and shorten healing time [14]. Conventional DFU management includes wound care, dressings, medication, off-loading, and glycemic control. Wound care consists of cleaning and debridement. To reduce bacterial growth and prevent further infection, cleaning the wound with saline or sterile water is recommended; on the other hand, debridement removes necrotic or infected tissue, which can impede wound healing [14]. Debridement may be performed surgically with sharp instruments, such as scalpels or scissors, to remove necrotic tissue, or non-surgically with enzymatic debridement using topical treatments such as creams and gels. Autolytic debridement uses enzymes to break down dead tissue, promoting tissue regeneration and reducing bacterial growth [35,36].

Dressings are used to protect the wound from contaminants, maintain a moist environment, and promote healing. Depending on the type of wound dressing, such as hydrogel, hydrocolloid, nanofiber films, and alginate dressings, it offers a distinct advantage, and the selection of dressing is based on wound depth and infection status [36]. However, traditional dressings primarily provide wound protection rather than promote angiogenesis or tissue regeneration. In DFU treatment, medication plays an important role; to manage infection, a topical antimicrobial is used. At the same time, systemic antibiotics such as cephalexin, dicloxacillin, and clindamycin are commonly prescribed to control infections caused by *Staphylococcus aureus* [37].

Thus, antihyperglycemic medications such as metformin and glibenclamide are commonly prescribed to help regulate blood glucose, reduce inflammation, and promote tissue repair, which are important for wound healing [34,38]. These oral medications are still being explored as topical treatments to minimize side effects. Topical natural products, such as honey (antiseptic properties), turmeric (anti-inflammatory and antibacterial properties), and aloe vera, reduce inflammation and promote wound healing, which is being explored as an alternative treatment for DFU [38,39].

**Figure 1:**
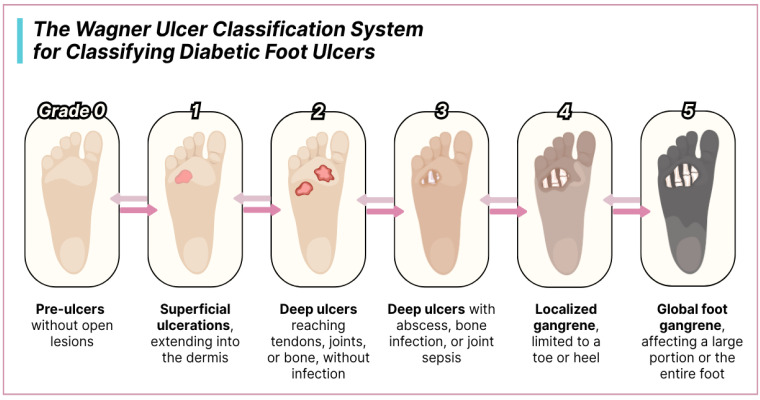
Wagner Scale of Classification of DFUs. Ulcer progression has six stages, which begin from pre-ulcerative lesions (Grade 0) and advance to deep ulcers (Grades 1–3), then to localized and extensive gangrene (Grade 4–5).

Advanced treatments such as negative-pressure wound therapy (NPWT) and hyperbaric oxygen therapy (HBOT) are being offered in addition to the standard care. NPWT removes exudates, reduces bacterial growth, and promotes wound closure [40]. On the other hand, HBOT promotes cell proliferation, reduces inflammation, and stimulates angiogenesis [41]. Despite promising results in improving healing rates and reducing amputation rates, both therapies are costly and not widely accessible [42]. The Wagner Ulcer Scale system, also known as the Meggitt-Wagner classification system, consists of six ulcer grades to assess the severity of the ulcer. It ranges from pre-ulcer lesions (Grade 0) to extensive gangrene (Grade 5), which would require amputation [43]. Additionally, the Wagner scale guides clinical treatment based on the wound type. For grades 0–2, it can be managed with standard care; for grades 3–5, it would require surgical or advanced technology treatment. The Wagner Ulcer Scale system for DFU severity is illustrated in Figure 1. 

Despite the current approach to DFU care improving patient outcomes, many obstacles remain. The complicated nature of diabetic wounds is not often addressed by conventional therapy, especially when there is poor angiogenesis or a serious infection [7]. Antimicrobial resistance (AMR) is exacerbated by the misuse of systemic antibiotics, which makes infection control more difficult [17]. The expense of treatment and the strain on patients are increased by frequent dressing changes and debridement [44]. Additionally, conventional moist wound dressings (i.e., hydrogels and alginates) primarily provide passive protection rather than an active healing stimulus [36]. Even though the Wagner scale of classification helps determine wound severity, it still does not directly guide the therapeutic interventions necessary for each stage [43].

The IWGDF 2023 guidelines focus on vascular assessment, infection management, and multidisciplinary care [33]. It emphasizes specific wound characteristics, such as depth, infection status, exudate, and perfusion or ischemia, which are important for therapeutic decision-making. However, recommendations for oxidative stress and regenerative therapies remain limited, making complementary strategies necessary for managing NPEs [33]. This limitation highlights the need for alternative treatment strategies to address infection control, oxidative stress, and tissue regeneration. Further studies show that these gaps can be filled by NPEs and their bioactive compounds [38].

## 3. Natural Product Extracts (NPEs) for DFU Treatment

Animals, plants, microorganisms, and almost all living cells are sources of NPEs with potential for use in drug formulations [19,35,38,45]. Bioactive compounds in NPEs are usually categorized as primary or secondary metabolites, produced, respectively, for survival and in response to external stresses [46]. Of the two categories, secondary metabolites have received more attention in drug discovery due to their diverse structures that contribute to novel mechanisms of action [47]. Phenolic compounds, terpenoids, and alkaloids are examples of secondary metabolites used in defense, communication, and signaling. However, their abundance is directly affected by environmental conditions, such as soil type, climate, and season [47,48,49]. NPEs are typically isolated from tissues, fluids, or secretions of various organisms using extraction methods carefully selected based on target bioactivities. Conventional methods, such as decoction, maceration, and percolation, as well as non-conventional methods such as ultrasound-assisted extraction, supercritical fluid extraction, and microwave-assisted extraction, are being used. Pre-treatment methods, including washing and drying, as well as solvent selection, are also critical for the production of target bioactive compounds [50,51]. Further separation is achieved using common chromatographic methods, such as thin-layer, high-performance liquid, and column chromatography [52]. Isolating bioactive compounds provides a greater understanding of their mechanisms of action, and, in most cases, secondary metabolites exhibit multiple bioactivities [47]. The antimicrobial, anti-inflammatory, antioxidative, and wound-healing properties of these bioactive compounds make them relevant for treating chronic wounds, such as DFUs [38]. Natural products derived from plants and animals that target oxidative stress, infection, inflammation, and impaired angiogenesis, thereby promoting wound closure and tissue regeneration, are currently the focus of DFU research [53,54].

### 3.1. Plant-Derived Natural Products

Plant-derived natural products, depending on the plant source, are reported to have antimicrobial, anti-inflammatory, anti-oxidative, cell proliferative activities, and odor-preventing effects [55,56]. For example, due to its phenolic and flavonoid content, the hydroalcoholic extract of aloe vera (AVHE) has demonstrated antioxidant, antidiabetic, anti-inflammatory, and fibroblast-proliferation effects. AVHE accelerates wound healing by alleviating oxidative stress and preventing bacterial buildup; it also demonstrates potential anti-inflammatory properties, as it stabilizes the human red blood cell membrane [57]. The same properties were observed in the isolated extracellular vesicles of *Aloe saponaria* (AS-EVs). The wound healing property of the PEG-based precipitated AS-EVs promoted angiogenesis and stimulated the skin proliferation and migration of fibroblasts [58]. Conversely, extracts from Abelmoschus esculentus (okra) fruit, either squeezed or boiled, were formulated into a hydrogel for chronic diabetic wound healing. Results revealed that Gel-MA okra hydrogels exhibit antioxidant activity and promote angiogenesis and cell migration. Boiled okra loaded in GelMA exhibited the best collagen fiber deposition, faster angiogenesis, and skin regeneration, which are attributed to its bioactive compounds: phenolic acids, flavonoids, terpenes, and other nutrients [59].

Furthermore, the curcuminoids in the methanolic extracts of the Ryudai gold variety of *Curcuma longa* have been explored [20]. Curcuminoids, specifically curcumin, demethoxycurcumin (DMC), and bisdemethoxycurcumin (BDMC) present in the plant, reduced the edema induced in rat paws significantly, indicating anti-inflammatory properties. Curcuminoids have also demonstrated antibacterial, antioxidative, and anti-diabetic properties [21]. On the other hand, a gel formulated with the total glycosides of *Centella asiatica* combined with nitric oxide, or *Centella asiatica* total glycosides nitric oxide gel (CATGNOG), has been reported to accelerate wound healing [24]. The results show that CATGNOG tested on rabbits showed no toxic effects on the skin and accelerated wound healing in diabetic rats within 14 days. Similarly, a combination targeting the miRNA-21-5p/TGF-β1/SMAD7/TIMP3 pathway was tested for its wound-healing effect. It used HaCat cells, in which the combination of asiaticoside and nitric oxide stimulated their migration and proliferation via the mentioned pathway. Hence, asiaticoside-nitric oxide promotes wound healing in diabetic wounds [25].

*Moringa oleifera* leaf extracts, on the other hand, exhibited antibacterial properties against *P. aeruginosa* and methicillin-resistant *S. aureus* on diabetic wounds in rat models. The extract also stimulated gene expression of VEGF and TGF-β, indicating its antioxidant and proliferative properties [60]. Similarly, properties from the *Moringa oleifera* hydro-alcoholic (MOHE) bark extract exhibited strong efficacy against diabetic Wistar rat wounds [61]. In addition, Sambong oil loaded into electrospun cellulose acetate nanofibers exhibits biocompatibility and antibacterial properties (against *E. coli* and *S. aureus*) and provides sufficient moisture on the wound surface [62]. When sambong components caryophyllene, jasmoline, and borneol are formulated into a gel, it demonstrates effective anti-inflammatory, antibacterial, and wound healing properties [63]. Moreover, *Origanum vulgare* L. also has antioxidant and antimicrobial properties [64]. The antimicrobial and accelerated wound-healing effects in rat wounds are attributed to carvacrol, the main active ingredient of *O. vulgare* [65].

The leaves of *Gliricidia sepium* have been formulated into a nanohydrogel and applied to diabetic rat wounds. The main bioactive compounds in the leaves, such as kaempferol, protocatechuic acid, and apigenin-7-O-glucoside, are responsible for the anti-inflammatory and antimicrobial properties of *G. sepium* leaves. When combined with zinc oxide nanoparticles in a PVA hydrogel, the controlled release enhances tissue regeneration and reduces cell apoptosis [66]. The leaves of *Psidium guajava* exhibit similar properties, as evidenced by testing the ethanolic leaf extract (PGELE) on diabetic male Wistar rats. The PGELE has demonstrated antioxidant, anti-inflammatory, antibacterial, granulation tissue formation, and neovascularization [67]. Combined *P. guajava* L. pada Tikus and *Melastoma malabathricum* L. dan increases wound healing, has antibacterial properties, and reduces exudates [68]. These properties are attributed to compounds such as quercetin, steroids, saponins, tannins, and flavonoids.

### 3.2. Animal-Derived Natural Products

Bees and snails are profound sources of natural products for DFU treatment. Various studies have already used honey to treat human DFUs, strengthening claims of its anti-inflammatory, antimicrobial, antioxidant, and odor-eliminating effects [69,70,71,72]. Medical-grade honey (MGH) has been used in treating DFU patients [69]. It has demonstrated the ability to combat infection and reduce exudate secretion, thereby improving healing and reducing wound odor. In particular, tropical honey was found to be more effective for Wagner grade 1 DFUs [71]. Although derived from the same source as honey, propolis is a different compound processed by bees and contains caffeic acid. An ethanolic extract of propolis-loaded PLGA nanoparticles on diabetic rats demonstrates the vascularization, fibroblast proliferation, and granulation tissue regeneration [22]. Knowing these individual properties of honey and propolis, both were combined into an ointment and applied to diabetic rat models [73]. The combination enhanced wound healing by promoting vascularization and fibroblast proliferation.

Additionally, snail mucus, which contains proteins and glycosaminoglycans, can inhibit the production of inflammatory cytokines, making it beneficial for diabetic wound healing. The snail mucus could also act as a natural adhesive due to its intertwined cationic proteins and anionic polymers [74]. A hydrogel based on a glycosaminoglycan inspired by snails has been shown to accelerate wound healing in diabetic mice. This property is attributed to the hydrogel’s anti-inflammatory effect, which promotes the transition of macrophages to the M2 type [75]. Table 1 summarizes the NPEs used in chronic wound or DFU treatment.

The current section demonstrates the therapeutic potential of NPEs in DFU due to their broad therapeutic properties, including antioxidant, anti-inflammatory, antimicrobial, angiogenic, and pro-healing effects [38,55,57]. However, existing limitations hinder clinical translation, primarily due to inconsistent NPEs. For instance, the standardization of bioactive concentrations is highly dependent on the plant source, growing conditions, and extraction techniques [10,26,27,56]. Seasons, location, and preparation techniques can also affect the plant extract and bee-derived materials, making reproducing challenging and introducing inconsistency [56,72,76,77]. Furthermore, processing, isolating, and stabilizing active compounds often require complex and costly purification processes [26,78]. Thus, the number of samples must be controlled, as overharvesting plants or using animal-based materials such as beeswax or snail mucin can harm ecosystems and raise ethical concerns [79]. However, the bioactive compounds extracted from NPEs, such as honey and essential oils, are sensitive and degrade in the presence of light, heat, and oxygen [28,80].

These are a few of the reasons why only a few of these treatments have been tested in large human trials. While animal and laboratory studies often appear promising, the lack of robust clinical evidence slows approval and wider use [81]. Safety must also be ensured in using these agents, as some people develop allergic reactions or skin irritation from NPEs such as essential oils, propolis, or aloe vera [82]. These qualities continue to be hindrances to NPEs’ potential for DFU care. Addressing these would require rigorous quality control, efficient extraction procedures, and innovative formulation techniques [10,83,84,85]. Table 2 summarizes the advantages and limitations of NPEs used in DFU treatment. 

## 4. Topical Medications for DFU Treatment

Managing DFUs with topical medications promotes faster wound healing, reduces malodor, and reduces infection severity. With a range of medications, from antibacterial to herbal formulations, their effects are being explored in advanced application delivery systems, such as hydrogels, nanofibers, and bandages infused with medication. This section explains the benefits of each type of medication, which can achieve much improved outcomes when combined with a natural plant.

### 4.1. Cephalexin

Cephalexin belongs to the cephalosporin class of β-lactam antibiotics that bind to penicillin-binding proteins [90]. It is commonly prescribed to treat soft-tissue and skin infections caused by *Staphylococcus aureus*, including the methicillin-resistant strain (MRSA). A topical application of cephalexin incorporated into poly(3-hydroxybutyrate-co-3-hydroxyvalerate) (PHBV) nanofiber is reported to exhibit significant antibacterial properties against MRSA that promote wound healing. The nanofiber also demonstrated high water uptake, a favorable biodegradation rate, and no signs of toxicity in vitro and in vivo cells [91].

Similarly, a 3D nanofibrous dressing consisting of PCL/Collagen loaded with Doxycycline hyclate and cephalexin shows good antibacterial activity against *E. coli* and *S. aureus*. It improves fibroblast proliferation, antibacterial activity, and collagen deposition. Due to its high porosity, good water absorption, biocompatibility, and antibacterial properties, it promotes faster wound healing [92]. Furthermore, studies have shown that a single dressing application is more effective for wound healing than multiple dressing changes.

Density Functional Theory (DFT) and Monte Carlo simulations have been used to evaluate the interactions in conventional cephalexin and graphene oxide-polyethylene glycol nanocomposites. The result shows a stable polyethylene glycol configuration with a yield of −25.67 kcal/mol, indicating strong absorption and a favorable active drug release in a moist environment rather than in a gaseous environment [93]. This highlights the potential of a graphene oxide-based composite as a drug-delivery substrate for cephalexin in wound treatment. Despite promising findings for cephalexin as a topical treatment, its effectiveness remains dependent on the delivery system, and the current evidence is limited to preclinical studies. A clinical trial is required to further validate the safety of the formulated dose for long-term wound-healing treatment in patients with DFU.

### 4.2. Mupirocin

Mupirocin, a topical antibiotic ointment that inhibits isoleucyl-transfer RNA, which obstructs the bacterial protein and RNA, causing cell death. This topical antibiotic is recognized globally for its efficacy in treating MRSA [94]. An application of nanohybrid systems containing mupirocin with selenium nanoparticles in a chitosan-based hydrogel shows significant enhancement of antibacterial activity against MRSA in a diabetic rat model, with improved wound contraction [95]. In addition, reductions in the inhibitory concentrations of mupirocin, selenium nanoparticles, and chitosan-based materials would highlight the nanohybrid system’s potential for therapeutic wound healing.

### 4.3. Neomycin

Neomycin, an aminoglycoside-based antibiotic, is used to treat and prevent bacterial infections, including those caused by Gram-negative bacteria [40,96]. The application of a combination therapeutic formulation containing honey, neomycin, and bacitracin in treating diabetic wounds shows significant improvement in antibacterial properties for wound healing, as tested in diabetic mouse models [97]. Using a 1:9 ratio with Cicatrin, the honey provided the best treatment against vancomycin-oxacillin-resistant *S. aureus* (VORSA). demonstrating a good wound contraction and size reduction with no signs of infections on the models as the combination of agents is administered, and found that the formulation is stable and spreadable. It shows that the healing properties of honey and medicinal plants are effective, promoting wound healing and reducing the risk of infection.

### 4.4. Metformin

Metformin, a biguanide hypoglycemic agent, is commonly used to treat non-insulin-dependent diabetes and reduces intestinal glucose absorption [98]. It has been shown to promote cellular proliferation and anti-apoptosis by increasing collagen I and III [99]. In diabetic conditions, hyperglycemia increases p53 and C-jun levels, and metformin counters this effect by accelerating wound healing, increasing collagen production, and reducing AP-1 expression. It shows that topical application of metformin reverses the effects that cause diabetes, thereby increasing the wound-healing rate and improving the repair process.

Furthermore, a combination of medications, including metformin, pioglitazone, and glibenclamide, has been incorporated into a chitosan–polycaprolactone–polyvinyl pyrrolidone nanofibrous scaffold for the treatment of diabetic wounds [100]. The multi-drug treatment has resulted in a significant acceleration in wound healing for type 1 diabetics. The combination treatment resulted in better skin regeneration and reduced cell infiltration and edema. As they observed in L929 at day 14, hair follicles had formed with this combination medication treatment.

### 4.5. Glibenclamide

Glibenclamide, a hypoglycemic agent in the sulfonylurea class, is commonly used by patients with type 2 diabetes to control their blood sugar levels [101]. Glibenclamide-loaded polymeric micelles have been evaluated for their efficacy in wound-healing applications [102]. Glibenclamide-loaded wound dressing was studied in rat models, where it was discovered that it has good cytocompatibility and promotes wound healing, and from the rats tested, the ratio that resulted in the fastest wound closure is the glibenclamide-loaded polyvinylpyrrolidone ratio of 1:0 (GB-SP/PVP1-0) and glibenclamide-loaded polyvinylpyrrolidone ratio of 2:1 (GB-SP/PVP 2-1).

This section emphasizes the therapeutic potential of this medication for DFU management, highlighting its targeted antimicrobial, antidiabetic, and anti-inflammatory properties that address the pathophysiology of persistent wounds. Antibiotics are widely studied for their potential to prevent bacterial growth, enhance fibroblast activity, and promote collagen deposition when delivered via advanced drug delivery systems [91,95,97]. Hypoglycemic medications demonstrate wound-healing benefits beyond glycemic control, including apoptosis reduction, stimulation of angiogenesis, and acceleration of wound closure when applied topically [99,102]. Despite the promising outcomes, clinical translation remains limited due to the stability of the medication, controlled kinetics release, and various wound conditions. Although various formulations have shown great potential in animal models, only a few human trials have been conducted. Additionally, combination therapy raises concerns about safety, efficacy, and dosage for long-term patient care. This limitation emphasizes the importance of strict standards, drug delivery methods, and clinical trial planning to further validate the long-term safety and effectiveness. Table 3 summarizes the advantages and limitations of selected agents as documented in existing studies.

## 5. Drug Delivery Systems for DFU Treatment

Beyond selecting therapeutic agents, the delivery method plays a critical role in managing DFU. Systems such as microneedles, bandages, hydrocolloids, hydrogels, and nanofibers are used to improve wound contact, control drug release, and enhance localized therapeutic effects, whether from natural plant extracts or conventional medications.

### 5.1. Microneedles

Microneedles (MNs) form microscale channels in the skin that allow drugs to be delivered directly to the target site. Their performance relies on design factors such as needle length, tip shape, and array density, ensuring that they penetrate the skin and can release control [105]. Biodegradable polymers such as hyaluronic acid (HA), chitosan, polyethylene glycol (PEG), and gelatin are commonly used to reduce skin irritation and degrade in a controlled manner, unlike less controllable materials such as metal and glass [104,106].

MNs composed of materials such as GelMA/PEGDA improve both mechanical strength and flexibility while maintaining adhesion to wound surfaces and sustaining drug release [106]. Depending on the intended application, MNs can be designed to dissolve, detach, or swell after insertion. This allows for the delivery of antibiotics, growth factors, oxygen, or nanoparticles at a specific stage of wound healing [107]. Drug release from MNs, like passive diffusion, matrix breaking down, and stimulus-responsive systems, can be triggered by local cues such as pH, glucose levels, or temperature. By responding to these cues, like pH or glucose, MNs can be adapted for different stages of wound healing. Their effectiveness, however, relies on careful design, including needle geometry, material selection, and release mechanisms. MNs are usually fabricated through micromolding, photolithography, or 3D printing using biodegradable polymers [108]. These methods allow more precise control over MN characteristics, which then improve their therapeutic potential.

### 5.2. Bandages

Recent developments in bandage design now integrate responsive biomaterials that can adjust to wound conditions, giving antimicrobial activity and also helping angiogenic support, besides the passive protection that conventional dressings give. A biocompatible bandage using supramolecular photothermal nanoparticles (MCC/CS NPs) can help to remove drug-resistant bacteria and also accelerate wound healing when it is activated by near-infrared laser light [109].

A bionic double-layer bandage developed is designed to replicate the natural structure of skin. It is designed with a combination of an antibacterial collagen top layer and a dermal collagen glycosaminoglycan scaffold that mimics dermis, helping support cell growth and antimicrobial activity [78]. A closed-loop bioelectronic bandage, on the other hand, is made to fit closely to the foot surface and monitor wound biomarkers such as pH, temperature, and oxygen levels [110]. Sensors that are embedded in this bandage can detect signs of infection or hypoxia and then trigger actions wirelessly, such as giving medication or delivering electrical stimulation.

Advanced bandages are made using techniques such as electrospinning, 3D bioprinting, or freeze drying. These techniques ensure that the bandages’ structure is breathable, multifunctional, and stable, as it also affects how well the bandage delivers drugs, adapts to skin shapes, and allows oxygen exchange, which is important for proper wound healing [84].

### 5.3. Hydrogels

Hydrogels create a moist, biocompatible environment that supports oxygen diffusion, cell migration, and tissue regeneration. Their high water content and porous structure make them well-suited for absorbing exudates while facilitating gas and nutrient exchange [111]. In addition, hydrogels can be engineered for controlled degradation, reducing the need for frequent dressing changes and sustaining oxygen diffusion and cell migration as they gradually biodegrade without removal [109].

Stimuli-responsive hydrogels release drugs in response to local changes such as elevated glucose, low pH, or excess ROS, enabling targeted delivery that helps reduce acidity, oxidative stress, and inflammation. Designed with reversible covalent bonds, these hydrogels degrade in a controlled manner and adapt to the needs of wounds, minimizing the need for frequent dressing changes [112].

Injectable hydrogels that use chitosan and β-cyclodextrin polymers are designed to deliver cinnamaldehyde [103]. When injected, it forms a solid protective layer that can penetrate stubborn biofilms and remove *S. aureus* and *P. aeruginosa* bacteria by 99.99% [106]. This method targets infection control and biofilms, making injectable hydrogels a solution for early Wagner scale DFUs. Hydrogels are formed via chemical crosslinking, ionic gelation, or thermal gelation. Polymer concentration and temperature affect the swelling ratio, porosity, integrity, and degradation [113].

### 5.4. Nanofibers

Nanofiber wound dressings mimic the extracellular matrix, resulting in cell attachment, proliferation, and differentiation. One approach involved the development of the bilayer electrospun nanofibers that use chitosan/polyvinyl alcohol (CS/PVA) and sodium alginate/polyvinyl alcohol (SA/PVA) blends, and the CS/PVA layer is loaded with Deferoxamine to help promote angiogenesis, while the SA/PVA layer serves as a protective barrier [114]. The PVA improved electrospinning process, with the addition of glutaraldehyde crosslinking, provided stability in moist wound conditions.

**Figure 2:**
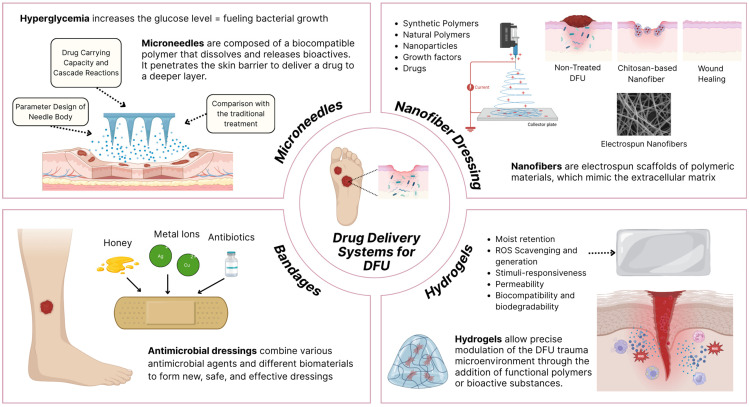
Advanced drug delivery systems for DFU treatment. Microneedles (**top left**) penetrate the skin for targeted drug delivery; nanofiber dressings (**top right**) mimic the extracellular matrix and support cell growth; antibacterial dressings (**bottom left**) incorporate antimicrobial agents (e.g., metal ions, honey) to control infection; hydrogels (**bottom right**) maintain a moist, biocompatible environment for wound healing. Each system offers distinct advantages depending on the wound characteristics.

Chitosan nanofibers have been shown to release N-acetyl-D-glucosamine and growth factors, such as PDGF and TGF-β, which contribute to reduced scarring [115]. Electrospinning produces these nanofibers by drawing fine threads from a liquid polymer, creating breathable mats that adhere effectively to wounds and replicate the structure of natural skin. Coaxial electrospinning allows controlled dual-phase drug release while additives such as bioglass or VEGF-mimetic peptides enhance cell growth. Natural compounds such as plant extracts, essential oils, or metal nanoparticles can be incorporated to support healing [116,117]. The advanced DDSs are illustrated in Figure 2 based on recent reviews of hydrogels, nanofibers, microneedles, and smart bandages for DFU treatment.

The effectiveness of DFU therapies, therefore, depends not only on the pharmacological agent but also on the delivery platform, which must ensure stability, controlled release, and patient safety. Each system presents unique strengths and limitations that should be matched to wound characteristics and patient needs. With various topical drug delivery technologies improving DFU wound management, their usefulness still hinges on balancing stability, efficacy, and safety. Topical agents show promising therapeutic effects, including antibacterial activity, antidiabetic properties, and accelerated wound healing [91,100]. Each delivery system, however, carries distinct advantages and limitations, such as maintaining drug stability, controlling release kinetics, and the risk for adverse effects like skin irritation [93,103]. Moreover, combinations of medications are often required to address the complex pathophysiology of DFUs, which makes formulation stability and safety profiles even more challenging [17,104].

Although recent advances in topical drug delivery, including nanofiber-based systems and stimuli-responsive hydrogels, show promise in improving targeted delivery while maintaining favorable safety profiles [107,109], optimal formulations must still be tailored to wound characteristics and patient-specific factors [85,93]. Microneedles, for example, may lack sufficient penetration depth in necrotic wounds, while hydrogels can degrade prematurely in high-exudate environments [106,112]. Table 4 summarizes the key advantages and limitations of these applications, highlighting how material selection, fabrication techniques, and drug-loading methods are significant factors in patient outcomes.

## 6. Combination Therapies

The overlapping pathologies of DFU could be addressed by combination therapies that target multiple pathways simultaneously. The broad bioactivity of NPEs combined with the advantages of potency and predictability of conventional drugs may result in three types of interactions: synergistic, additive, or antagonistic. The goal is to leverage the individual characteristics of agents and formulate a synergistic (enhanced effect) or additive (summed effect) interaction. An antagonistic or reduced effect is an interaction to be avoided when combining NPEs and conventional drugs and integrating them into DDSs [118]. Understanding these interaction types is essential for designing rational combination therapies. The following sections present specific examples and the biological mechanisms they influence in DFU treatment.

### 6.1. Sambong Leaf Extract in Aloe-Infused Nanofiber Bandages

The combination of *Blumea balsamifera* (Sambong) leaf extract with *Aloe vera* in an electrospun nanofiber dressing integrates two complementary mechanisms: Sambong provides antimicrobial and antioxidant activities [62], and Aloe vera keeps moisture and supports fibroblast proliferation [119]. When incorporated in cellulose acetate nanofiber mats (fiber diameter approximately 450–600 nm), Sambong oil has shown good NIH-3T3 cell survival, biphasic release kinetics, optimal moisture vapor transmission rate (MVTR) at 1750–2450 g/m^2^/day for wound breathability, antioxidant activity, and antibacterial efficiency against *E. coli* and *S. aureus*. These are evaluated by Fourier Transform Infrared Spectroscopy (FTIR) and X-ray diffraction (XRD) tests, and are in vitro data [28]. With these results, it may be possible to enhance tissue restoration by combining antibacterial oil with a moisture-retaining nanofiber scaffold.

Aloe vera has been separately incorporated into chitosan, PCL, and keratin nanofibers, with 90 nm-thick fiber shells. Results report increased cell adhesion and proliferation without having any harmful effects. In addition, the fibers were shown to have drug-eluting properties, thereby qualifying them for continuous therapeutic release [120]. In an existing patent, US20130337090A1 [121], the formulation approach closely resembles other well-known aloe vera-based wound treatment methods. Aloe vera-based applications and the patent’s method for developing bioactive, plant-based dressings are conceptually compatible, especially since both use natural substances to promote and replicate the body’s inherent healing mechanisms [121]. However, no single study has directly tested Sambong oil and Aloe vera in a nanofiber system. The individual evidence suggests that such a hybrid dressing could provide both oxidative stress reduction and a moist environment beneficial for wound healing. A direct experimental study of this specific combination is needed.

### 6.2. Propolis Incorporated with Charcoal-Infused Bandages

Propolis, which has strong antimicrobial and anti-inflammatory properties [23], and activated charcoal, which has a high capacity to adsorb bacteria, toxins, and wound exudate, have the potential to work in tandem to improve infection control and manage the wound environment in dressings [122,123].

Propolis alone has shown promise in DFU patients. In a randomized controlled trial involving 60 patients with septic DFUs, 76.6% of the propolis group achieved full wound healing within 3 weeks, compared with 0% in the control group. Early granulation tissue formation, as well as significant decreases in ulcer surface area and depth, bacterial counts, inflammatory indicators, dressing-related discomfort, and wound odor, were also observed. However, the authors noted certain limitations, including the relatively small sample size (*n* = 60) and the short follow-up period (3 weeks only). Long-term studies are also needed for validation despite promising results [124]. In another randomized, placebo-controlled clinical trial, a 3% propolis spray applied over 8 weeks in 31 patients with type 2 diabetes showed greater connective tissue deposition and an average reduction in wound area of 4 cm^2^. Local improvements in antioxidant and anti-inflammatory status, including increased glutathione (GSH) and GSH/GSSG ratios (*p* < 0.02), decreased tumor necrosis factor-α (TNF-α) levels, and increased interleukin-10 (IL-10) levels at the wound site were also observed. Assessments at both the macro- and microscopic levels confirmed improved wound-healing properties [88]. With this, further studies with larger sample sizes and longer follow-up periods are needed to confirm that these findings establish long-term efficacy.

As for activated charcoal, it was combined with baicalein and incorporated in multifunctional chitosan fiber-based wound dressings fabricated via screen printing. A high MVTR of about 2570 g/m^2^/day and an air permeability of around 1837 mm/s were recorded from the dressings, which contribute to a moist yet breathable environment. The dressings exhibited strong antibacterial activity, inhibiting the growth of over 95% of *S. aureus* and *E. coli*, as well. The activated charcoal component effectively absorbed malodorous gases, with deodorization rates of 84% for ammonia and 75% for acetic acid. In cytotoxicity testing, 90% of the human dermal fibroblasts survived after 48 h, indicating good biocompatibility [85].

Moreover, a luminous pH-sensing bandage designed for wound diagnostics incorporates activated charcoal. The charcoal was used in a microencapsulation system to help retain and stabilize pyranine, a pH-sensitive fluorescent dye, on cationic polymeric microparticles. The calcium-alginate hydrogel containing these microparticles formed a biocompatible and secure bandage that could be applied directly to wounds. In both lab and animal tests, the bandage has a consistent fluorescent response across a pH range of 6 to 9 (R^2^ = 0.9909). Preventing dye leakage and achieving accurate pH readings can be achieved with charcoal, which retains over 99% of the dye in biological fluids after 24 h. Since wound pH is closely linked to healing, this charcoal-based sensor bandage could offer a simple, non-invasive way for clinicians to monitor wound healing in real time [83]. These individual characteristics of activated charcoal, combined with propolis, have not yet been directly tested. Hence, the proposed combination remains conceptually promising but requires substantial evidence.

### 6.3. Algae-Derived Hydrogel Infused with Silver Nanoparticles

Ulvan, a green algal-derived sulfate-rich polysaccharide, can be used to create hydrogels that are highly biocompatible and retain high moisture [125]. Particles in the 1–100 nm size range, known as silver nanoparticles, are prized for their broad antimicrobial properties [126]. By infusing Ulvan with silver nanoparticles, the combination offers high moisture retention and antimicrobial properties, making it a viable option for wound healing.

Ulvan-based hydrogel films (UHF) are developed by crosslinking Ulvan with boric acid and incorporating glycerol as a plasticizer. Results show good swelling capacity (82% to 130% in 1 h), water vapor transmission rate (WVTR) from 1856 to 2590 g/m^2^/24 h, strong antioxidant activity reducing hydroxyl radicals, and broad-spectrum antibacterial activity against *S. aureus*, *P. aeruginosa*, *E. coli*, and *S. epidermidis*. A porous structure was observed in scanning electron microscope (SEM) images, supporting gas exchange. These findings support the potential of the films as versatile wound dressings [125]. When silver nanoparticles were incorporated in UHF, creating UHF-AgNPs, the physical properties, such as pH, viscosity, swelling capacity, and water vapor transmission rate, were similar between the two formulations (UHF–AgNP0.5 and UHF–AgNP1). The nanoparticles were mostly spherical, with an average diameter of less than 35 nm and good stability, as indicated by a zeta potential of −24 mV. AgNPs can disrupt bacterial membranes and generate oxidative stress, confirming their potential for effective antimicrobial treatment [127]. However, in vivo testing was not conducted, and these studies include only in vitro characterization tests.

### 6.4. Copper-Infused Nanofiber Wound Dressings Enhanced with Propolis Extract

Copper nanoparticles facilitate angiogenesis and collagen synthesis [128]. Combining copper nanoparticles with propolis and its phenolic components [129] will provide an alternative to wound dressings. Recently, the successful fabrication and characterization of nanofibrous mats composed of cellulose ester (CE) and copper oxide-decorated CE (Cu/CE) have been achieved. For wound healing, the mats’ MVTR were optimal at 1900–2100 g/m^2^/day and exhibited strong antibacterial activity against *S. aureus* and *E. coli*, with greater potency at higher copper concentrations. In the first 24 h, a quick, controlled release of Cu is observed, followed by a steady release over the next six days. High biocompatibility (≥82% viability) was demonstrated by cell viability tests, suggesting that these mats have the potential to be used as safe and effective antibacterial dressings for wounds [130,131].

Through the combined effects of photothermal therapy and copper ion release, (Cu^2+^@PTPU-15) copper ions embedded in a photothermal polyurethane nanofiber wound dressing have been shown to efficiently disrupt *P. aeruginosa* and *S. aureus* biofilms, demonstrating excellent antibiofilm effectiveness. It demonstrated strong angiogenic activity, promoted fibroblast migration, exhibited biocompatibility, enhanced collagen deposition and tissue regeneration, decreased bacterial load, and sped up wound closure in an infected rat wound model. Additionally, it helped move the wound from an inflammatory to a healing phase by modulating the immune response, increasing anti-inflammatory IL-10 levels, and decreasing pro-inflammatory TNF-α [132]. However, these studies tested copper alone, and the proposed combination remains conceptually promising but has not been directly evaluated in any wound model. Direct testing is needed to confirm additive or synergistic effects.

### 6.5. Microneedle Systems Loaded with a Combination of Propolis and Oregano Essential Oils

**Figure 3:**
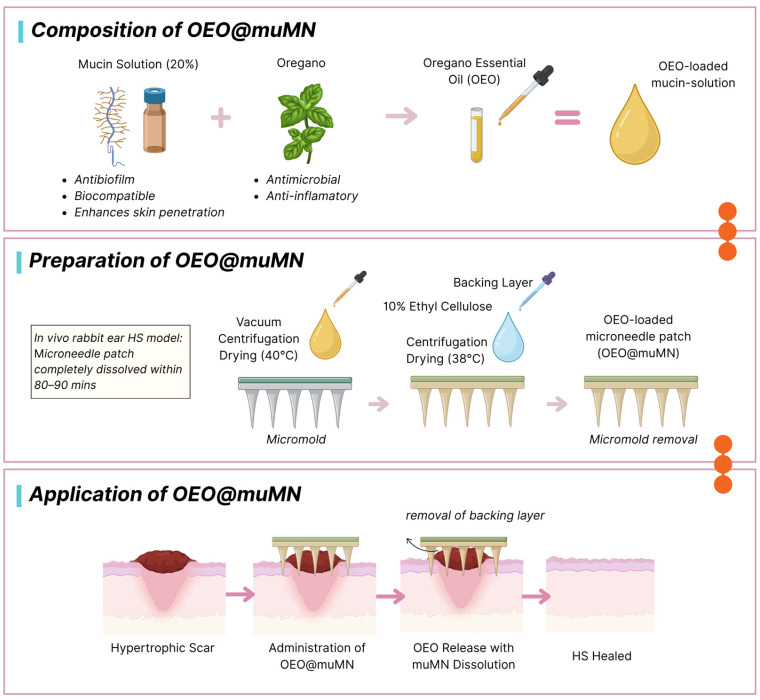
Compositions, Preparation, and Application of OEO@muMN to Hypertrophic Scar Model. Oregano essential oil (OEO) combined with mucin solution is processed into microneedles with an ethyl cellulose backing layer to form OEO@muMN. When applied to hypertrophic scars, mucin dissolution enables controlled OEO release, promoting scar healing.

A dissolvable microneedle (muMN) patch incorporating the combination of mucin and oregano essential oil (OEO) was fabricated through a two-step micromolding process. For flexibility and structural stability, a thin ethyl cellulose support layer was added. The biodegradability of this system and its effectiveness were confirmed in preclinical in vivo studies using rabbit ears, which demonstrated that the microneedles were well tolerated and fully dissolved within 80–90 min. Upon dissolution, the microneedles release OEO directly into the deeper layers of the skin, enabling penetration of scar tissue. This localized delivery enhances drug retention at the wound site, reduces biofilm formation, and accelerates wound healing without the need for repeated dressing changes. Its high therapeutic potential, ease of setup, and compatibility with natural chemicals make it a treatment option for upcoming clinical trials and practical use in managing DFUs [89]. However, the microneedle system has not been evaluated with propolis incorporated, and testing in diabetic animals or in clinical trials has not yet been performed. Figure 3 shows the fabrication and mechanism of this proposed muMN patch.

### 6.6. Synergistic Potential of Centella asiatica, Poloxamer, and Zinc Oxide Nanocomposite

**Figure 4:**
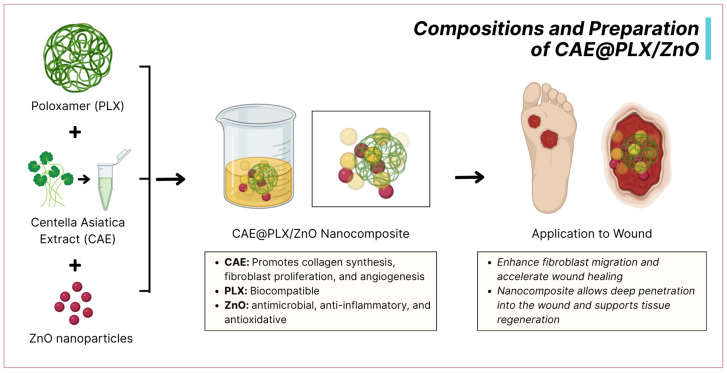
Compositions and Preparation of CAE@PLX/ZnO. CAE@PLX/ZnO is created by combining poloxamer (PLX), zinc oxide nanoparticles (ZnO NPs), and *Centella asiatica* extract (CAE). Green represents PLX, yellow represents CAE, and red represents ZnO NPs. This nanocomposite supports enhanced wound healing by improving tissue regeneration and antimicrobial activity when applied to wound models.

A nanocomposite material, CAE@PLX/ZnO, combines *Centella asiatica* extract (CAE), poloxamer (PLX), and zinc oxide nanoparticles (ZnO) to improve wound healing, combat bacterial infections, reduce oxidative stress, and eliminate chronic inflammation. The antibacterial activity of ZnO nanoparticles has been confirmed by the presence of inhibition zones against *S. aureus* and *P. aeruginosa*. On the other hand, CAE was confirmed to downregulate pro-inflammatory cytokines (IL-1β, IL-6, IL-8) while upregulating anti-inflammatory cytokines (IL-4, IL-10), thereby creating a conducive environment for healing. Shown in Figure 4 is the preparation and mechanism of the CAE@PLX/ZnO nanocomposite.

The CAE@PLX/ZnO nanocomposite leverages the complementary mechanisms of the three agents. CAE promotes collagen synthesis, fibroblast proliferation, and angiogenesis, which accelerates wound closure. PLX serves as a biocompatible carrier with thermo-reversible properties, allowing the active compounds to be released in a controlled manner. Because of its self-assembling nature, the stability and delivery of ZnO and CAE to the wound site improve. Lastly, ZnO provides antimicrobial and anti-inflammatory effects and supports tissue regeneration.

The small size (<50 nm) of the CAE@PLX/ZnO nanocomposite can penetrate deeper into the wound bed, enhancing its overall effectiveness. It also helps reduce ROS levels, reducing oxidative stress and supporting the healing process. In vitro scratch assay with L929 fibroblasts revealed enhanced cell migration and proliferation. Furthermore, the nanocomposite promotes angiogenesis, as evidenced by improved vascularization in the HUVECs tube formation assay. Finally, the nanocomposite has also facilitated collagen gel contraction, mimicking the natural wound contraction process in diabetic rat wound models [86]. Although in vitro and in vivo preclinical tests exist for this combination, quantitative and clinical data would provide more evidence of the claimed synergistic potential.

### 6.7. Origanum vulgare Essential Oil and Moroccan Thyme Honey Mixture

Combining *Origanum vulgare* essential oil (OEO) with Moroccan thyme honey (MTH) treats inflammation and bacterial infections. This is attributed to the phenolic acids and flavonoids in MTH, as well as thymol and carvacrol in OEO. The in vivo testing of this mixture included the Carrageenan-induced edema and the trauma-induced edema. Across the three mixtures of different extracts, OEO-MTH shows the best results, with 51.65% inhibition at 3 h in the first test and 52.81% inhibition in the second test. The study then concludes that the OEO-MTH mixture is beneficial for treating chronic wounds, including DFUs, and has huge potential for clinical trials and applications [133]. However, evidence is limited to acute inflammation models and not in diabetic or infected wound models. The combination may be promising, but it requires further validation.

### 6.8. Quantitative Synergy of Polyphenolic Extracts and Cefixime

Synergy or interaction between two agents is quantified using the fractional inhibitory concentration (FIC) indices. In a study, methanol, aqueous, and ethyl acetate extracts from nine medicinal plants are tested in combination with the antibiotic cefixime against resistant clinical isolates of *S. aureus*, *E. coli*, *K. pneumoniae*, *Acinetobacter*, and *P. aeruginosa*. The medicinal plants included *Murraya koenigii*, *Terminalia chebula*, *Curcuma amada*, *Momordica charantia*, *Chamomilla recutita*, *Mentha longifolia*, *Nigella sativa*, *Gentian lutea*, and *Terminalia arjuna*.

RP-HPLC analysis confirmed the presence of significant quantities of polyphenols (gallic acid, quercetin, cinnamic acid, luteolin) that are known to disrupt bacterial membranes and inhibit efflux pumps. Results of the checkerboard assays indicate that most of the combinations yielded FIC indices ≤0.5 (total synergy) and 0.5–0.75 (partial synergy). For instance, *C. amada* methanol extract combined with cefixime showed total synergy against *S. aureus* and *K. pneumoniae*, resulting in a 4- to 8-fold reduction in MICs. Time-kill kinetics has also indicated concentration and time-dependent bactericidal activity. Moreover, protein leakage assays indicated that membrane damage was the mechanism. Overall, this study provides a validated methodology for quantifying synergy between natural extracts and conventional antibiotics. This approach is important in future combination studies for DFU management [134].

### 6.9. Methodological and Translational Challenges of Combination Therapies

All in all, Section 6.1, Section 6.2, Section 6.3, Section 6.4, Section 6.5, Section 6.6, Section 6.7 and Section 6.8 discussed combinations ranging from conceptually proposed systems to those with direct experimental validation. Several combinations (e.g., propolis-charcoal, Centella-ZnO) are supported by in vitro or in vivo data, but few have progressed to clinical evaluation, and most studies used only healthy or non-diabetic animal models. Furthermore, the formulation stability, scalability, regulatory pathways, and drug interaction in combination therapies remain largely unexplored. Therefore, most combinations in this study are inferred from separate studies of individual components rather than directly tested as integrated systems. Even when tested, quantitative measures such as FIC indices are rarely reported, making true synergy difficult to confirm [118,134].

Aside from the lack of direct testing, there is a risk of antagonistic interactions, where plant compounds reduce or counteract drug effects [118]. For instance, side effects from flavonoids and terpenoids may arise because they can interfere with drug metabolism through cytochrome P450 enzymes or efflux transporters [118,135]. Moreover, translating these combinations into advanced DDSs can be costly and technically complex, limiting their application in resource-constrained settings [136]. A recent systematic review also emphasized that poor standardization and inconsistent extraction methods undermine reproducibility and clinical applicability of reported synergies [118]. Table 5 summarizes the main strengths and limitations of the reviewed combinations and highlights the evidence gaps that future research must address.

## 7. Synthesis

DFUs involve multiple factors based on their complex pathophysiology. This includes chronic inflammation, impaired angiogenesis, infection, and oxidative stress. While antibiotics, debridement, and dressings–conventional care are still the clinical standard, limitations with accessibility, efficacy, and resistance still exist. The IWGDF guidelines on managing DFUs recommended using rating systems prior to therapy to personalize treatment. However, classification systems that proliferated for decades, including the Wagner, University of Texas, SINBAD, and WIfI systems, had reduced complex wounds to a single grade or score [33,34]. All fail at the same task: guiding treatment selection.

A Wagner Grade 3 ulcer may represent deep soft tissue infection, abscess, osteomyelitis, or joint sepsis. These are four conditions requiring four different interventions [33]. A SINBAD score of 3 can arise from any combination of site, ischemia, neuropathy, infection, area, and depth [33,34]. Composite scores obscure the clinical variables that actually determine therapy. Therefore, the updated 2023 IWGDF guidelines suggest describing the individual variables that compose the systems rather than a total score [34]. This review rejects the staging paradigm and instead organizes combination therapies based on four key wound characteristics that guide clinical decisions: depth, infection status, exudate level, and perfusion. Table 6 presents a proposed framework that aligns NPEs and conventional drugs with these specific wound characteristics. This framework is intended to guide future study design, not immediate clinical decision-making.

The selection of the combinations is based on wound characteristics as mentioned. These wound characteristics signal the bioactivities required for healing. Depth of the wound corresponds to angiogenesis and cell proliferation, exudate levels to antioxidant and anti-inflammatory action, infection status to antimicrobial therapy, and perfusion to angiogenic or vasodilatory support [137,138].

Depth determines the type of DDS to be used, such as that superficial wounds require a different DDS than deep wounds. Microneedles are ideal for superficial and biofilm-infected DFUs because they deliver activities to the viable epidermis where biofilm forms [89,107]. Meanwhile, injectable hydrogels are necessary for deep wounds with tunneling or irregular geometry, as they conform to the wound bed and provide sustained release in hard-to-reach spaces [86,103]. Nanofibers can serve both purposes, but electrospinning parameters must be adjusted to achieve the desired thickness and porosity based on the wound depth [115,116].

On the other hand, the presence and intensity of infection guide the antimicrobial strategy. For wounds without established infection, prophylaxis is necessary to prevent biofilm formation. Aloe-Sambong nanofibers provide antioxidant and anti-inflammatory activities that could damage the wound environment before bacteria colonize [28,119]. Localized infections may be treated with propolis-oregano microneedles, which help target established biofilms. Carvacrol in oregano damages bacterial membranes even at sub-MIC concentrations, while flavonoids in propolis inhibit quorum sensing and efflux pumps [23,89]. This combination attacks infection through multiple mechanisms, potentially slowing the emergence of resistance. For DFUs with suspected biofilm, higher doses and sequential release are required. Coaxial nanofibers can release an initial burst of membrane-disrupting agents that penetrate biofilms, followed by sustained release of antibiotics to maintain their effect [115,117]. This strategy has been shown with synthetic antibiotics but has not been applied yet to NPEs [132].

Depending on the exudate levels, fluid management can also be adjusted. Hydrogel sheets provide hydration while allowing gas exchange, making them suitable for low exudate wounds [111,119]. For moderate exudates, materials with both absorptive and antimicrobial properties are needed. Charcoal-based bandages, for example, can absorb bacteria, toxins, and breakdown products, while also helping reduce odor, which has been reported to have removal rates of 84% for ammonia and 75% for acetic acid [85]. Propolis adds sustained antimicrobial and anti-inflammatory activity. The combination addresses both infection and exudate simultaneously. Lastly, high exudating wounds require maximum fluid handling. A study on copper-propolis nanofibers achieved WVTR of 1900–2100 g/m^2^/day, which is sufficient for heavily draining wounds and still provides sustained copper release for angiogenesis [131]. Silver-ulvan hydrogels have a similar absorptive capacity with a broader antimicrobial activity [125,127].

Furthermore, perfusion status supports the need for angiogenesis. Ischemic wounds cannot heal without neovascularization, and antimicrobial bandages alone are insufficient. Centella-metformin combinations may suffice for this since Centella upregulates VEGF and TGF-β via the miRNA-21-5p pathway [25,86], while metformin reduces hyperglycemia-induced apoptosis and promotes collagen synthesis [99]. Delivered in thermo-responsive hydrogels, these agents provide sustained angiogenic stimulation in the hypoxic wound environment [86]. Moringa-metformin nanofibers also offer an alternative approach, in which moringa upregulates VEGF and TGF-β and provides antimicrobial activity against MRSA [60], while metformin supports the metabolic environment for healing [100]. This combination may be particularly valuable when infection and ischemia coexist.

The inflammatory response is also an important determinant of healing outcomes. While acute inflammation is essential, prolonged dysregulated inflammation leads to excessive scarring or non-chronic healing wounds. NPEs like honey, curcumin, and aloe vera aid in downregulating pro-inflammatory mediators, reducing ROS, and promoting an anti-inflammatory (M2) macrophage phenotype [138]. Combinations like aloe-sambong (prophylaxis) and propolis charcoal (infection control), where the NPE contributes anti-inflammatory activity that complements the other agent’s function.

The proposed combinations provide multiple mechanisms to address the complex pathophysiology of DFUs. However, challenges remain in ensuring drug stability, standardizing natural extract compositions, and scaling up production, particularly in resource-limited environments. In light of these findings, further investigation is warranted to evaluate the strategic integration of natural compounds and delivery technologies into current clinical protocols [137]. Important factors and suggestions to encourage this integration and guide future advancements in DFU treatment are outlined. The framework established in this study is intended as a hypothesis-generating tool rather than a clinical guideline. Evidence levels range from in vitro to human pilot studies; however, most combinations have not been tested as combinations in any wound model. FIC indices are also absent for most antimicrobial pairs, and optimal ratios are unknown [118,134]. There are also no present head-to-head comparisons that indicate the best combination for a certain wound characteristic. Overall, the compatibility of these combinations within specific DDS, including factors such as shelf life, storage, and bioactivity retention, remains underexplored. Because of this, the framework presented is based on current evidence and should be seen as a guide for future research rather than a validated clinical approach.

## 8. Conclusions

DFUs are a persistent global health challenge characterized by complex wound conditions, including ongoing infection, oxidative stress, impaired angiogenesis, and chronic inflammation that delays healing. These pathologies do not exist individually but as coexisting impairments that current treatments address one at a time. This review argues that combination therapy, enabled by advanced DDSs, offers a realistic path to multitargeted intervention.

The study highlighted combinations of natural bioactive compounds, including curcumin, aloe vera, honey, and propolis, and conventional agents like cephalexin and metformin, to develop more effective, multi-targeted therapies, by organizing them not by the flawed composite scores, but by the wound characteristics recommended by IWGDF: depth, infection status, exudate level, and perfusion. This provides clinicians and researchers with a mechanism-based roadmap for rational combination selection.

Given the preclinical nature of most cited evidence, three priorities emerge for further investigation. Firstly, propolis-charcoal bandages, having the strongest backing (a human RCT for propolis and strong in vitro evidence for charcoal), should be evaluated in clinical trials for moderately exudative, infected Grade 2-equivalent ulcers. On the other hand, propolis-oregano microneedles show compelling preclinical results for superficial and biofilm-positive ulcers but require diabetic animal studies followed by phase I trials. Lastly, centella-ZnO nanocomposites address poor angiogenesis in ischemic ulcers, but require in vivo validation before clinical consideration.

Obstacles to clinical adoption persist and must be addressed. Standardization of natural products through marker-based quality control, ensuring stability in DDSs through optimized formulation science for each combination, and regulatory navigation through hybrid drug-botanical pathways are some of the methods future studies need to prioritize. Moreover, cost, usability, manufacturing, scalability, and patient adherence must be prioritized from the outset. Without addressing these concurrent problems, promising combinations will remain in the literature rather than reaching patients. Emerging artificial intelligence and machine learning may be employed in natural product screening and wound-healing data analysis, enabling accelerated compound discovery, identification of relevant biomarkers, and support for the creation of individualized treatment plans.

Classification systems will continue to evolve, such that the Wagner scale may fade, and SINBAD or WIfI may dominate. Fundamental wound characteristics (depth, infection, exudate, and perfusion), however, will remain constant. Based on the framework on these individual features, DFU ensures its relevance and addresses the complex pathophysiology. This has the potential to reduce hospital stays, lower the risk of infection and amputation, improve patient mobility and independence, and ultimately reduce the long-term healthcare burden.

## Figures and Tables

**Table 1:** NPEs, their compounds, and their role in DFU treatment.

Natural Product Extract	Bioactive Compound/s	Role in DFU	Evidence Level	References
*Aloe barbadensis* *Miller* (Aloe vera)	Alkaloids, tannins, flavonoids, phenols, and sterols	Antioxidant, anti-inflammatory, antimicrobial, and fibroblast proliferation	II	[57]
*Abelmoschus* *Esculentus* (Okra)	Flavonoids, phenolic acids, and terpenes	Antioxidant, antibacterial, anti-inflammatory, wound healing, and angiogenesis	II	[59]
*Curcuma longa* (Turmeric)	Curcuminoids, alkaloids, flavonoids, sterols, and terpenoids	Antimicrobial, antioxidant, anti-inflammatory, anti-diabetic, and wound healing	II	[20,21]
*Centella asiatica*	Glycosidic asiaticoside, madecassoside, asiatic acid, flavonoids, and phenolic acids	Antibacterial, antioxidant, anti-inflammatory, and wound healing	II	[25,26]
*Moringa oleifera* (Malunggay)	Steroids, alkaloids, flavonoids, phenolic acids, and tannins	Cell proliferation, antioxidant, and antimicrobial	II	[60]
*Blumea balsamifera* (Sambong)	Flavonoids, volatile oil	Antioxidant, antibacterial, and fibroblast proliferation	II	[62]
*Origanum vulgare* (Oregano)	Flavonoids, phenolic acids, carvacrol, and essential oils	Antioxidant, antimicrobial, and inhibitory activities	II	[64]
*Gliricidia sepium* (Madre de Cacao)	Phenolic acids, kaempferol, and flavonoids	Antimicrobial, anti-inflammatory, and wound healing	II	[66]
*Psidium guajava* (Guava)	Tannins, saponins, flavonoids, steroids, and phenolic quercetins	Antimicrobial, antioxidant, anti-inflammatory, and wound healing	II	[68]
Honey	Hydrogen peroxide, flavonoids, and phenolic acids	Antioxidant, angiogenesis, antibacterial, anti-inflammatory, and VEGF stimulant	I	[72]
Propolis	Flavonoids, esters, aldehydes, and caffeic acid	Antibacterial, antioxidant, anti-inflammatory, and wound healing	I	[22]
Snail mucus	Glycosaminoglycan and proteins	Anti-inflammation, cell proliferation, antimicrobial	II	[74]

Note: Evidence Level ranges from Level I to Level IV, indicating the type of data. Level I: Human clinical studies, including randomized controlled trials or observational studies. Level II: Preclinical animal (in vivo) studies of diabetic or related wound models.

**Table 2:** Advantages and limitations of natural products used in DFU treatment.

Aspect	Advantages	Limitations	Evidence Basis	References
Therapeutic Effects	Multiple bioactivities; Less toxic than synthetic drugs	Effects heavily depend on source, season, and extraction method	Preclinical studies (in vivo and in vitro)	[22,26,28,38,55,56,57]
Source and Availability	Widely available; Sustainable in raw form with traditional use backing	Risk of overharvesting and ecological impact; Source variability affects consistency	Observational and review articles	[44,55,62,84]
Formulation and Stability	Compatible with hydrogels and nanofibers; Combinations possible for enhanced effects	Degrades with light, heat, or oxygen; Volatile oils and proteins have a short shelf life	In vitro and formulation studies	[22,26,28,62,86,87]
Safety	Generally biocompatible; Suitable for topical use	Risk of skin irritation, allergic reactions (propolis, essential oils); Potential drug interactions (CYP450 interference)	Human studies (clinical trials, case reports) and in vitro	[22,57,88,89]
Cost and Accessibility	Affordable and locally available in raw form; Potential for community-based sourcing	Not yet scalable in low-resource environments; Costly processing and integration	Economic analyses cited by review articles	[22,55,62,84,87]

Note: Evidence Basis indicates the primary types of studies supporting the statement. This includes preclinical, in vitro, and human studies from the selected references.

**Table 3:** Advantages and limitations of topical medications used in DFU treatment.

Medication	Advantages	Limitations	Evidence Level	References
Cephalexin	Strong anti-MRSA activity; Promotes healing and collagen deposition; Non-toxic nanofiber suitable for moist environments; No signs of toxicity in vivo and in vitro	Less effective in dry environments; Requires advanced delivery systems (PHBV nanofiber, PCL Collagen nanofiber, PEG composite)	II	[90,91,92,93]
Mupirocin	High antibacterial activity and improves wound contraction; Sustained release in chitosan hydrogel; Promotes tissue regeneration	Sensitive to oxidation and aggregation; Particles can be unstable, requiring a precise dosage to avoid toxicity	II	[94,95]
Neomycin	Target Gram-negative and Gram-positive bacteria; Tissue regeneration, angiogenesis, and anti-inflammatory effects	Can mimic infection, delay healing, and increase risk	II	[96,97]
Metformin	Increases collagen I/III; Reduces hyperglycemia markers; Inhibits anti-apoptosis; Enhances skin repair with Pioglitazone and Glibenclamide	More effective in combination; Primarily used as a hypoglycemic agent	II	[98,99,100]
Glibenclamide	Shows good cytocompatibility; Effective wound closure in specific polymer ratios	Limited clinical studies; Require precise formulation for best results	II	[101,102,103,104]

Note: Evidence Level ranges from I to IV, indicating the type of data discussed in the sections. Level II: Preclinical animal (in vivo) studies using models of diabetic or related wounds.

**Table 4:** Advantages and limitations of drug delivery systems used in DFU treatment.

Drug Delivery System	Advantages	Limitations	Evidence Basis	References
Microneedles	Direct drug access to tissue; Customizable release; Biodegradable materials	Limited penetration; Possible skin irritation or incomplete delivery	Preclinical studies (in vitro, ex vivo, animal models)	[104,105,106,107,108]
Smart Bandages	Can monitor biomarkers and act automatically	Complex design; Limited access in low-resource settings	Preclinical (animal models) and proof-of-concept studies	[78,84,109,110]
Hydrogels	Maintains moisture, absorbs exudates; Some offer smart, stimuli-responsive drug release	May lack structural integrity in high-exudate wounds; Frequent reapplication needed	In vitro and animal studies; few clinical evaluations	[103,106,109,111,112,113]
Nanofibers	Mimics natural tissue; Promotes healing via sustained bioactive release	Require crosslinking agents or additives for stability; Handling can be delicate	In vitro and animal studies; early clinical trials	[114,115,116,117]

Note: Evidence Basis indicates the primary types of studies supporting the statement. This includes preclinical, in vitro, and human studies from the selected references.

**Table 5:** Advantages and limitations of combinations used in DFU treatment.

Combinations	Advantages	Limitations	Evidence Level	Evidence Basis	Reference
Sambong leaf extract integrated into aloe-infused nanofiber bandages	Antioxidant, antimicrobial, angiogenic Controlled release, breathable, biocompatible	Allergy risk and a few clinical trials	IV	Inferred from separate studies: Animal and human (Aloe vera) + In vitro (Sambong)	[28,62,87,119,120,121]
Propolis incorporated with charcoal-infused bandages	Antimicrobial and anti-inflammatory	Allergy risk and variable propolis composition	IV	Inferred from separate studies: Human pilot study (propolis) + In vitro (charcoal)	[83,85,88,122,123,124]
Algae-derived hydrogel infused with silver nanoparticles	Broad antibacterial and antioxidant properties; Moist environment; Oxygen exchange; Eco-friendly synthesis	Silver cytotoxicity risk; Nanoparticle instability; Limited clinical translation	III	Clinical Trials and animal (Ulvan/Silver) + In vitro (Ulvan)	[125,126,127]
Copper-infused nanofiber wound dressings enhanced with propolis extract	Collagen promotion and biofilm disruption; Sustained copper release; In vitro biocompatibility	Copper toxicity risk; High cost; Long-term safety unclear	IV	Inferred from separate studies: Human (Propolis) + In vitro (Copper)	[128,129,130,131,132]
Microneedle systems loaded with a combination of propolis and oregano essential oils	Antimicrobial and anti-inflammatory; Painless, targeted delivery, quick dissolution, and sustained release; Fewer dressing changes	Not clinically approved; Possible skin irritation; Oil stability issues	IV	Inferred from separate studies: Animal (Oregano); Propolis not included in combination	[89]
Synergistic potential of *Centella asiatica*, Poloxamer, and Zinc Oxide Nanocomposite	Enhances fibroblast migration and angiogenesis; Anti-inflammatory and antimicrobial; Controlled release via thermo-responsive; ROS scavenging and wound contraction	Complex formulation; ZnO toxicity risk; Limited human data	II	Direct testing of combination in vivo (diabetic rat)	[86]
*Origanum vulgare* Essential Oil and Moroccan Thyme Honey mixture	Strong anti-inflammatory and antibacterial synergy; Reduces edema, accelerates healing; Natural pH/moisture inhibits bacteria	Allergy risk; Oil instability; No controlled release system	III	Direct testing of combination in vivo (non-diabetic)	[133]
Quantitative Synergy of Polyphenolic Extracts and Cefixime	Quantified synergy (FIC ≤ 0.5); Time-dependent antibacterial activity; Validated methodology for synergy testing	In vitro only and non-DFU model; Not tested in DDSs	III	Direct testing of combination in vitro checkerboard assays with clinical isolates	[134]

Note: Evidence Level ranges from I to IV, indicating the type of data discussed in the sections. Level II: Preclinical animal (in vivo) studies using models of diabetic or related wounds. Level III: In vitro studies using cell lines and bacterial assays or in vivo non-diabetic models. Level IV: Conceptual or inferred (not directly tested as a system and based on separate studies of individual components).

**Table 6:** Combination framework based on wound characteristics.

Wound Characteristic	Clinical Assessment	Recommended Combination	DDS	Rationale	Evidence Level	Evidence Basis	References
Depth	Superficial (subcutaneous)	Propolis + oregano	Dissolving microneedles	Microneedles penetrate biofilm; essential oils disrupt membranes; propolis inhibits efflux pumps	IV	Inferred from separate studies: Animal (Oregano); Propolis not included in combination	[89]
	Deep (tendon/ligament/bone)	Centella + ZnO	Injectable hydrogel	Hydrogel conforms to irregular cavities; Centella drives VEGF; ZnO provides antimicrobial	III	Directly tested in vivo (diabetic rat)	[86]
Infection	No infection (prophylaxis)	Aloe + sambong	Nanofiber mat	Anti-inflammatory + antioxidant prevents biofilm formation	IV	Inferred from separate studies: Animal and human (Aloe vera) + In vitro (Sambong)	[28,62]
	Localized infection	Propolis + oregano	Microneedles	Synergistic antimicrobial activity	IV	Inferred from separate studies: Animal (Oregano); Propolis not included in combination	[89]
	Biofilm suspected	Propolis + oregano (high dose)	Coaxial nanofibers	Core–shell allows sequential release; first dose disrupts biofilm, second maintains activity	IV	Conceptual, inferred from separate studies	[89,115]
Exudate	Low/minimal	Aloe vera	Hydrogel sheet	Provides moisture without maceration	III	In vitro	[112]
	Moderate	Propolis + charcoal	Multilayer bandage	Charcoal adsorbs exudate and toxins; propolis controls infection	IV	Inferred from different studies: Human (propolis) + in vitro (charcoal)	[79,117]
	High	Copper + propolis	Cellulose nanofibers	High WVTR (1900–2100 g/m^2^/day); sustained copper release	IV	Inferred from separate studies: Animal (Oregano); Propolis not included in combination	[123,124,125]
Perfusion	Adequate	Any combination	As appropriate	-	-	-	-
	Ischemic or low perfusion	Centella + metformin	Thermo- responsive hydrogel	Centella upregulates VEGF; metformin reduces apoptosis; hydrogel releases at body temperature	IV	Inferred from different studies: In vitro (metformin) + Animal (Centella)	[80,99]
		Moringa + metformin	Nanofiber scaffold	Moringa upregulates VEGF/TGF-β; metformin supports collagen synthesis	IV	Conceptual, inferred from separate studies	[60,94]

Note: Evidence Level ranges from I to IV, indicating the type of data discussed in the sections. Level III: In vitro studies using cell lines and bacterial assays or in vivo non-diabetic models. Level IV: Conceptual or inferred (not directly tested as a system and based on separate studies of individual components). Evidence Basis indicates the primary types of studies supporting the statement. This includes preclinical, in vitro, and human studies from the selected references.

## Data Availability

No new data were created or analyzed in this study. Data sharing is not applicable to this article.

## Abbreviations

The following abbreviations are used in this manuscript:

AMR	antimicrobial resistance
CAE	*Centella asiatica* extract
DDS	drug delivery system
DFU	diabetic foot ulcer
DM	diabetes mellitus
IWGDF	International Working Group on the Diabetic Foot
MIC	minimum inhibitory concentration
MRSA	methicillin-resistant *Staphylococcus aureus*
NPWT	negative-pressure wound therapy
NPEs	natural product extracts
ROS	reactive oxygen species
TGF-β	transforming growth factor beta
TNF-α	tumor necrosis factor alpha
VEGF	vascular endothelial growth factor
